# Guide Field Reconnection: Exhaust Structure and Heating

**DOI:** 10.1029/2018GL077670

**Published:** 2018-05-19

**Authors:** J. P. Eastwood, R. Mistry, T. D. Phan, S. J. Schwartz, R. E. Ergun, J. F. Drake, M. Øieroset, J. E. Stawarz, M. V. Goldman, C. Haggerty, M. A. Shay, J. L. Burch, D. J. Gershman, B. L. Giles, P. A. Lindqvist, R. B. Torbert, R. J. Strangeway, C. T. Russell

**Affiliations:** ^1^ The Blackett Laboratory Imperial College London London UK; ^2^ Space Sciences Laboratory University of California Berkeley CA USA; ^3^ LASP/Department of Astrophysical and Planetary Sciences University of Colorado Boulder Boulder CO USA; ^4^ Department of Physics and Institute for Physical Science and Technology University of Maryland College Park MD USA; ^5^ Department of Physics University of Colorado Boulder Boulder CO USA; ^6^ Department of Physics and Astronomy University of Delaware Newark DE USA; ^7^ Now at The Department of Astronomy and Astrophysics University of Chicago Chicago IL USA; ^8^ Southwest Research Institute San Antonio TX USA; ^9^ NASA Goddard Space Flight Center Greenbelt MD USA; ^10^ Department of Space and Plasma Physics Royal Institute of Technology Stockholm Sweden; ^11^ Space Science Center University of New Hampshire Durham NH USA; ^12^ Department of Earth, Planetary, and Space Sciences University of California Los Angeles CA USA

**Keywords:** Magnetic Reconnection, Magnetosheath, Plasma heating, Electron hole, Magnetospheric Multiscale

## Abstract

Magnetospheric Multiscale observations are used to probe the structure and temperature profile of a guide field reconnection exhaust ~100 ion inertial lengths downstream from the X‐line in the Earth's magnetosheath. Asymmetric Hall electric and magnetic field signatures were detected, together with a density cavity confined near 1 edge of the exhaust and containing electron flow toward the X‐line. Electron holes were also detected both on the cavity edge and at the Hall magnetic field reversal. Predominantly parallel ion and electron heating was observed in the main exhaust, but within the cavity, electron cooling and enhanced parallel ion heating were found. This is explained in terms of the parallel electric field, which inhibits electron mixing within the cavity on newly reconnected field lines but accelerates ions. Consequently, guide field reconnection causes inhomogeneous changes in ion and electron temperature across the exhaust.

## Key Points

- A guide field reconnection exhaust was encountered by MMS in the magnetosheath ~100 ion inertial lengths downstream from the X‐line
- A density cavity forms on one edge of the exhaust with embedded electron jetting toward the X‐line and electron holes on the cavity edge
- The parallel electric field causes electron cooling and ion heating in the cavity and inhomogeneous temperature profiles across the exhaust

## 1 Introduction

**Figure 1:**
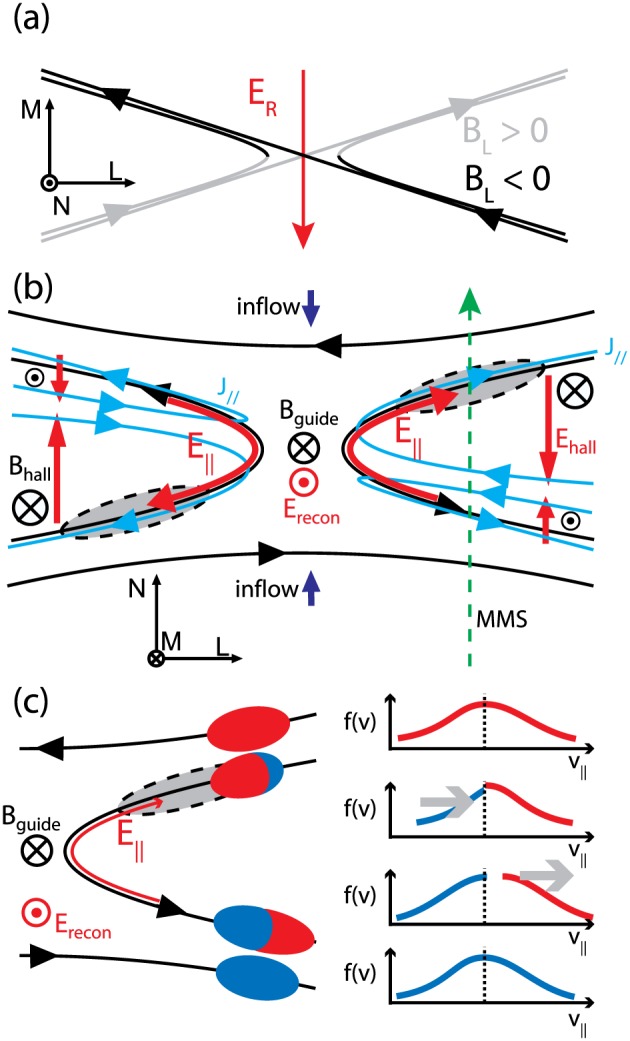
(a) Sketch of guide field reconnection viewed looking down onto the current sheet and (b) along the out‐of‐plane direction. The density cavity is shown in gray with electric fields in red and the current density in blue. (c) Sketch showing the role of the parallel electric field in modifying the distribution of electrons passing the midplane and the consequent change in density and temperature, described in detail in the text. MMS = Magnetospheric Multiscale.

Magnetic reconnection releases stored magnetic energy in the form of hot jets of plasma confined to the reconnecting current sheet (e.g., Fuselier & Lewis, 2011; Paschmann et al., 2013). In general, the reconnecting magnetic fields may not be antiparallel, and the addition of a guide field **B**_**G**_ changes the structure of the reconnection exhaust (e.g., Eastwood et al., 2013; Øieroset et al., 2016). The introduction of a parallel electric field, E_||_, causes electrons to move along the magnetic field leading to the formation of two cavities (Figures 1a and 1b), with a thickness of the order of *ρ*_*S*_ = (1/Ω_i_)(*T*_*e*_/*m*_*i*_)^0.5^ (the ion gyroradius based on the electron temperature); the ions undergo polarization drift across the field (Kleva et al., 1995; Pritchett & Coroniti, 2004). These cavities are predicted to play an important role in electron acceleration and are potentially a site for instabilities leading to electron hole formation (Cattell et al., 2005; Drake et al., 2003, 2005). The Hall field structure is also distorted in the presence of **B**_**G**_, due to the **J** × **B**_**G**_ force on the electron outflow (Eastwood et al., 2010; Horiuchi & Sato, 1997; Huba, 2005).

Although the magnetic field topology changes in the diffusion region, much of the energy release takes place in the exhaust, where the majority of the reconnecting plasma is processed through the exhaust edges. Reconnection naturally leads to the formation of counterstreaming populations in the exhaust, and so parallel heating is to be expected; for example, at the magnetopause it is found that Δ*T*_*i*,par_~2Δ*T*_*i*,perp_ (Phan et al., 2014). It has been proposed that **B**_**G**_ will reduce Δ*T*_*i*,par_ and that strong perpendicular ion heating will only “switch on” when the thickness of the exhaust boundary is sufficiently small, such that the particle gyroradius is larger than the boundary thickness and pick‐up behavior occurs. It can be shown that this occurs when the plasma beta is below some limiting value (Drake et al., 2009; Drake & Swisdak, 2014). Observations of *T*_*e*_ in magnetopause reconnection also find that the guide field may affect the temperature anisotropy in the exhaust with Δ*T*_*e*,perp_ essentially suppressed when B_G_ > B_R_, the reconnecting field component (Phan et al., 2013). No clear dependence of Δ*T*_*e*,par_ on B_G_ was found.

Novel high time resolution data from the Fast Plasma Instrument (FPI; Pollock et al., 2016) on Magnetospheric Multiscale (MMS; Burch et al., 2016) is now enabling the physics of guide field reconnection to be probed in new detail. In a symmetric guide field reconnection exhaust encounter ~12.5 ion inertial lengths (*d*_*i*_) from the X‐line (B_G_/B_R_ = 2), MMS resolved an asymmetric density profile, with a depletion filling one half of the exhaust followed by a density enhancement in the other half (Øieroset et al., 2016). An increase in *T*_*i*,par_ was found in conjunction with the density depletion, whereas *T*_*i*,perp_ was enhanced on the opposite side of the current sheet. In contrast, *T*_*e*,par_ only increased with the density enhancement.

Reconnecting current sheets in the solar wind provide an excellent opportunity to further study the structure of essentially symmetric reconnection exhausts with a variety of guide fields at a range of distances from the X‐line (Gosling, 2012; Gosling et al., 2005; Gosling & Phan, 2013; Mistry et al., 2015, 2016, 2017; Phan et al., 2010), and these larger‐scale current sheets are also observed in the magnetosheath (Øieroset et al., 2017; Phan et al., 2007; Wilder et al., 2017). There are also indications that magnetosheath reconnection occurs in the turbulent current sheets downstream of quasi‐parallel shocks (Retinò et al., 2007; Vörös et al., 2017; Yordanova et al., 2016). However, both the solar wind and the magnetosheath flow rapidly convect reconnecting current sheets over the observing spacecraft, and exhaust crossings may only last a few seconds, meaning that high time resolution MMS data are necessary to fully resolve their structure. For example, MMS has encountered a symmetric guide field reconnection exhaust passing near the electron dissipation region in the magnetosheath, resolving asymmetric Hall fields, a strong region of parallel electric field, parallel electron heating and electron phase space holes (Wilder et al., 2017).

Here we present new observations of guide field reconnection using MMS. The reconnecting solar wind current sheet was observed in the Earth's magnetosheath with a guide field B_G_/B_R_ = 0.7, and the spacecraft crossed the current sheet ~100 *d*_*i*_ from the X‐line, resolving the fine structure of the exhaust far from the X‐line. We examine both the exhaust structure and the ion and electron heating. The MMS data reveal that the heating is highly inhomogeneous and that in the edge cavity there is simultaneously electron cooling and enhanced parallel ion heating. This inhomogeneity is linked to the action of E_||_.

## 2 Data and Overview

The magnetosheath reconnection exhaust was observed on 21 January 2016 01:06:41.10–01:06:52.04 UT, at [8.2, −8.7, −1.1] Re (Earth radii) GSE (geocentric solar ecliptic). The ambient plasma was characterized by a magnetic field strength |*B*| ~ 64 nT, a relatively high number density *n* ~ 84 cm^−3^, ion temperature *T*_*i*_ ~ 160 eV, electron temperature *T*_*e*_ ~ 40 eV, and a total plasma beta *β* = 1.7. The inflow conditions on either side of the exhaust were stable for tens of seconds and largely symmetric. The maximum spacecraft separation of the four tetrahedrally arranged spacecraft was 14.7 km, less than the ion inertial length *d*_*i*_ = 24.9 km.

**Figure 2:**
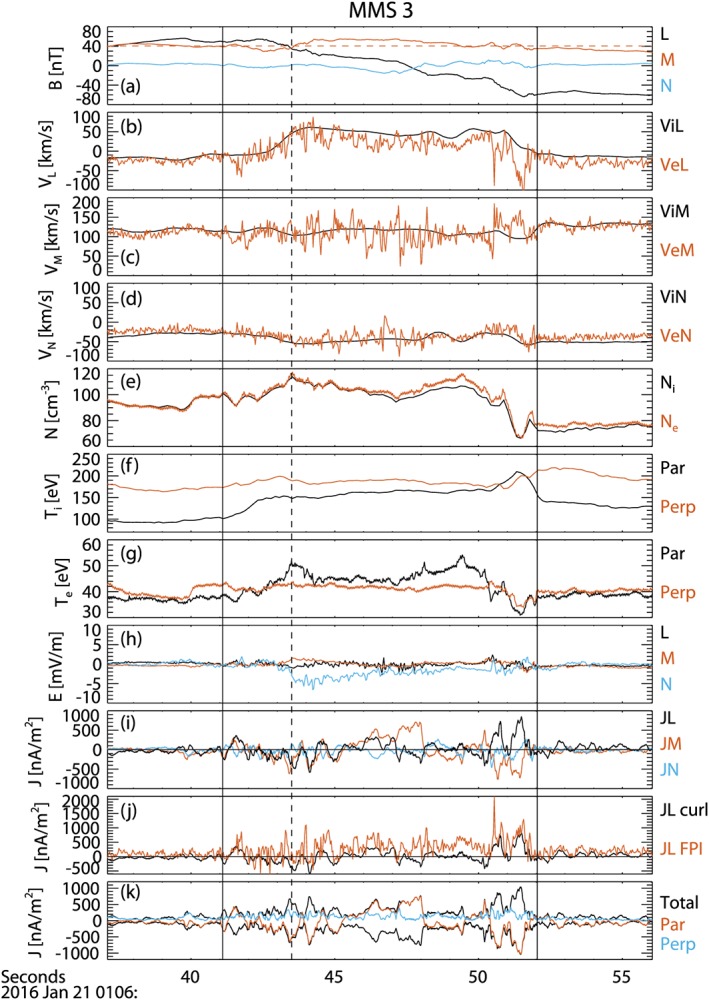
MMS3 observations of (a) magnetic field (red dashed line marks the guide field); (b–d) L, M, and N components of the ion (black) and electron (red) velocity; (e) ion and electron density; (f and g) ion and electron temperature; (h) electric field in the exhaust frame (the transformation velocity is the average ion flow velocity <*v* ≥ [−90.7, −78.3, −25.4] km/s in geocentric solar ecliptic); (i) current density derived using the curlometer technique; (j) L component of the current density using Fast Plasma Instrument particle measurements and the curlometer; and (k) parallel and perpendicular current density derived using the curlometer. MMS = Magnetospheric Multiscale.

Figure 2 shows MMS3 magnetic field data at 128 vectors/s (Russell et al., 2016), electron and ion moments at 30 and 150 ms, respectively (Pollock et al., 2016), and electric field data in the rest frame of the reconnection exhaust at fast survey mode data rate (32 vectors/s; Ergun et al., 2016; Lindqvist et al., 2016; Torbert et al., 2016), that is, where the electric field due to the magnetosheath flow, −**v**_**i**_ × **B**, in the inflow region has been subtracted from the measured electric field. The data have been rotated into a boundary normal coordinate system using hybrid minimum variance analysis (Gosling & Phan, 2013) applied to the interval 01:06:41.10–01:06:52.04 UT. **N** = [0.830, −0.522, −0.194] GSE, **M** = [−0.547, −0.830, −0.108] GSE, **L** = [0.105, −0.195, 0.975] GSE. The current sheet normal **N** = (**B**_**1**_ × **B**_**2**_)/|**B**_**1**_ × **B**_**2**_|, where **B**_**1**_ and **B**_**2**_ are average magnetic field vectors on either side of the interval. The guide field direction, **M** = **N** × **L′**, where **L′** is the maximum variance direction obtained from minimum variance analysis of the interval (Sonnerup & Scheible, 1998). The exhaust outflow direction, **L** = **M** × **N**. There is very close agreement between the four spacecraft on large scales and very similar coordinate systems are found; important differences between the spacecraft are mentioned below.

Between 01:06:41.10 and 01:06:52.04 UT (marked by solid vertical lines) B_L_ changes sign, and there is an increase in both *v*_*i*,L_ and *v*_*e*,L_ (Figures 2a and 2b) indicating a reconnection exhaust crossing (as shown in Figure 1b), since v_L_ is anticorrelated with B_L_ at the leading edge and correlated at the trailing edge (Gosling et al., 2005). The exhaust velocity is ~60 km/s, and the Alfvén speed based on the reconnecting field is C_A,L_ = 123 km/s; sub‐Alfvénic outflows are not uncommon in reconnection exhausts (e.g., Mistry et al., 2017). In guide field reconnection the component of the magnetic curvature vector in the **M** direction also leads to weaker out‐of‐plane flows that are oppositely directed either side of the current sheet. In this event, it is predicted there would be in the –v_M_ and +v_M_ perturbations at the leading and trailing edges of the exhaust; Figure 2c may show some tentative evidence for this.

MMS was located below the ecliptic plane with the jet predominantly oriented in the +*z*_GSE_ direction. This places the X‐line in the magnetosheath away from the magnetopause, and therefore, it is unlikely that the exhaust geometry would be influenced by the magnetopause. The exhaust crossing duration (10.9 s) and the ambient average magnetosheath v_N_ flow speed (−42.6 km/s) give an exhaust width of 465 km or 18.6 *d*_*i*_. The ambient v_L_ flow speed (−17 km/s) means that during the crossing, MMS may have moved approximately 7 *d*_*i*_ in the L direction away from the X‐line. Overall, a canonical reconnection rate of 0.1 therefore implies that the spacecraft were ~100 *d*_*i*_ downstream of the X‐line.

## 3 Exhaust Structure

Although the plasma density is enhanced in the exhaust relative to the surrounding inflow (Figure 2e), a cavity is observed at the end of the exhaust encounter. Its duration, 01:06:50.4–01:06:51.9 UT, corresponds to a width of 63.9 km = 2.6 *d*_*i*_ = 4 *ρ*_*S*_, and its location is consistent with theoretical expectations, being confined in a thin layer close to the separatrix where the parallel electric field points away from the X‐line (Figure 1b; Kleva et al., 1995; Pritchett & Coroniti, 2004). Compared to a previous observation of guide field reconnection at a distance of ~12 *d*_*i*_ from the X‐line (Øieroset et al., 2016), the density depletion in that event is on the same side of the exhaust as the present case but fills approximately half of the exhaust. This may suggest that the density cavity is limited in size; closer to the X‐line, it occupies more of the exhaust outflow.

Previous observations found no evidence for an electron flow toward the X‐line in the cavity at ~12 *d*_*i*_ from the X‐line (Øieroset et al., 2016). Here however, there is fast electron flow toward the X‐line within the cavity with *v*_*e*,L_ reaching −110 km/s, opposite to the overall bulk exhaust flow and the ion flow in the cavity. The flow is predominantly field aligned. This provides the first direct confirmation of the expected return electron flow in the cavity but reveals that it is considerably slower than the predicted maximum speed of the electron Alfvén speed (Pritchett & Coroniti, 2004).

Turning to the magnetic field observations, in the exhaust, B_M_ initially decreases from an average value of 40.5 nT (thus, B_G_/B_R_ = 0.7; magnetic shear = 110°) to 29 nT, before increasing to 55 nT (|ΔB_M_| ~ 15 nT; Figure 2a). This negative/positive perturbation to B_M_, with the reversal located at B_L_ = B_G_ (marked by the vertical dashed line), is the expected signature of the Hall magnetic field B_Hall_ (Figure 1b). B_M_ then remains enhanced through the reversal in B_L_, with some oscillatory structure at the end of the encounter where the density cavity was observed. Note that B_G_ perturbs the Hall field reversal away from the cavity (Figure 1b). B_Hall_ is accompanied by a normal electric field (E_N_), initially slightly positive but then negative throughout the majority of the exhaust, reaching −7 mV/m. This is consistent with the Hall electric field, predicted in simulations and illustrated in Figure 1b (Pritchett & Coroniti, 2004).

The variation in the out‐of‐plane magnetic field across the exhaust, ∂B_M_/∂N is associated with J_L_ where J_L_ ~ −∂B_M_/∂N + ∂B_N_/∂M. Figure 2i shows **J**_**Curl**_, the current derived from the four spacecraft magnetic field measurements using the curlometer technique (Robert et al., 1998). The negative gradient in B_M_ at the start of the exhaust encounter corresponds to a positive J_Curl,L_. J_Curl,L_ is then negative but filamented. This filamentation reflects the fact that the reversal in B_Hall_ is not in fact smooth. The curlometer calculation therefore shows that the Hall current density is structured and filamented on ion scales. Subsequently, J_Curl,L_ is large just prior to the cavity encounter and then within the cavity itself.

The current density can also be calculated using the FPI data directly where **J**_**FPI**_ = ne(**v**_**i**_ − **v**_**e**_), and the ion data are interpolated to the electron time resolution (Figure 2j). This reveals there are positive J_FPI,L_ spikes separating the negative J_FPI,L_ regions during the Hall field reversal. This filamentation and reversing of J_L_ is in fact seen at all four spacecraft with significant differences between the four spacecraft on occasion. This implies that in addition to distinct ion‐scale filamentary structure that is resolved by the curlometer, even smaller‐scale filamentation may also exist that is resolved by significant differences in the FPI measurements from individual satellites. This has been reported in other MMS observations at the magnetopause (Phan et al., 2016). In contrast, within the density cavity at the trailing edge of the exhaust, J_FPI,L_ is largely similar between the satellites and similar to J_Curl,L_. This implies that the cavity is not as filamented or structured below the ion scale.

## 4 Plasma Temperature Changes

The high time resolution MMS data allow exploration of the heating in much more detail. The ions undergo predominantly parallel heating, which is enhanced in the density cavity on the trailing edge of the exhaust (Figure 2f). The electrons also undergo predominantly parallel heating in the main exhaust, but there is a noticeable cooling in the cavity where both *T*_*e*,par_ and *T*_*e*,perp_ are reduced below the inflow temperature.

To make contact with previous analysis, we first consider the average change in the total ion temperature *T*_*i*_. Relative to the inflow region, Δ*T*_*i*_ = 16 eV in the exhaust and Δ*T*_*i*_ = 32 eV in the cavity. Observations both in the solar wind and at the magnetopause show that typically, Δ*T*_*i*_ = 0.13 *m*_*i*_C_A_^2^ (Drake et al., 2009; Phan et al., 2014). Here 0.13 *m*_*i*_C_A_^2^ = 20.9 eV, and so the bulk ion heating is comparable to previous studies. We next consider the anisotropic change in temperature, as discussed by Drake and Swisdak (2014). It is predicted that Δ*T*_*i*,par_ = m_i_C_A_^2^ B_L,in_^2^/(B_L,in_^2^ + B_M,in_^2^) = 107 eV for this event. The total plasma beta *β* = 1.7, larger than the predicted *β*_crit_ = 0.2, and so no perpendicular heating is predicted. Observationally, Δ*T*_*i*,par_ = 46 eV in the exhaust, Δ*T*_*i*,par_ = 94 eV in the cavity, and there is no clear evidence for perpendicular ion heating. Physically, to cause significant perpendicular ion heating, the change in E_N_ should occur on short‐length scales comparable to the ion motion at the edge of the exhaust. This is not observed; the strongest E_N_ is found deeper in the exhaust away from the cavity region where E_N_ is relatively weak and uniform.

**Figure 3:**
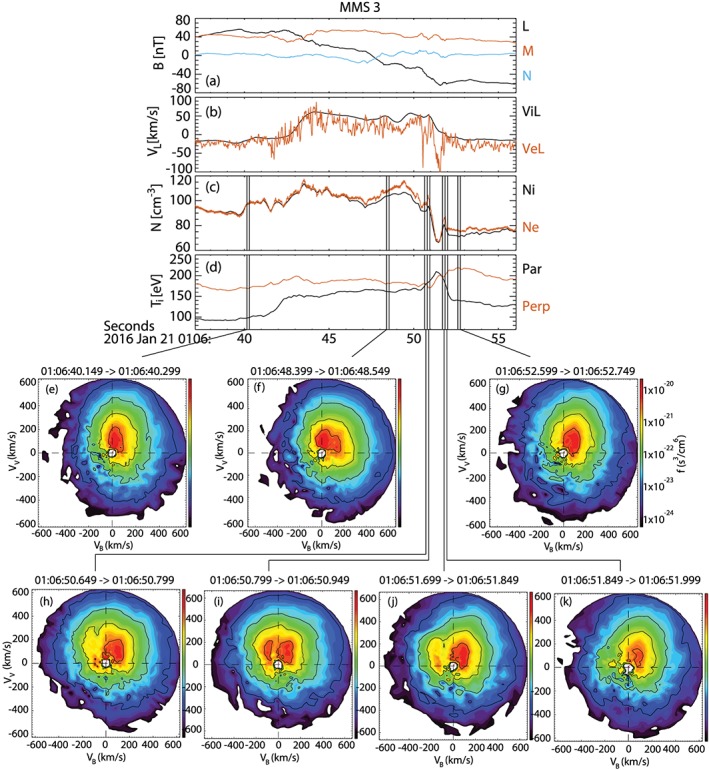
MMS 3 observations of (a) magnetic field, (b) L component of ion and electron velocity, (c) ion and electron density, (d) ion temperature, and (e)–(k) Fast Plasma Instrument ion distribution functions. Cuts in the plane defined by the magnetic field and the ion velocity are shown, taken at the times marked by vertical lines in panels (a)–(d). Note that (h) was measured just outside the cavity but in the exhaust and (i) was measured at the edge of the cavity 150 ms later. (j) and (k) were measured at the outer edge of the cavity 150 ms apart. MMS = Magnetospheric Multiscale.

Figures 3e–3g show examples of the ion distribution in the inflow region before the exhaust, in the exhaust itself, and in the trailing inflow region. Distributions are shown as cuts in the v‐b plane. Within the exhaust, counterstreaming beams are present (Figure 3f). Figures 3h–3k show that within the cavity, there is a very sharply confined ion beam moving antiparallel to the magnetic field. Referring to Figure 1b, these ions are moving away from the X‐line, parallel to E_||_. We conclude that the increase in *T*_*i*,par_ is due to the addition of this enhanced antiparallel streaming population and is presumably linked to acceleration by the parallel electric field associated with the cavity.

The change in total electron temperature Δ*T*_*e*_ can be similarly examined. In the Phan et al. (2013) observational study of magnetopause bulk electron heating, it was found that Δ*T*_*e*_ = 0.017 *m*_*i*_C_A,asym_^2^ where C_A,asym_ is the asymmetric inflow Alfvén speed. Relative to the inflow region, in this event Δ*T*_*e*,par_ = 5.6 eV, Δ*T*_*e*,perp_ = 0.5 eV, and Δ*T*_*e*_ = 2.2 eV averaged across the exhaust (but not including the cavity). Here 0.017*m*_*i*_C_A_^2^ = 2.7 eV and so the bulk electron heating is comparable to previous experimental observations. MMS shows that this heating is almost entirely parallel, which is again consistent with previous observations suggesting that perpendicular electron heating is suppressed when B_G_/B_R_ = 1 (Phan et al., 2013). In the cavity, Δ*T*_*e*,par_ = −6.5 eV, Δ*T*_*e*,perp_ = −6.0 eV, and Δ*T*_*e*_ = −6.1 eV. Thus, the cooling is approximately isotropic.

**Figure 4:**
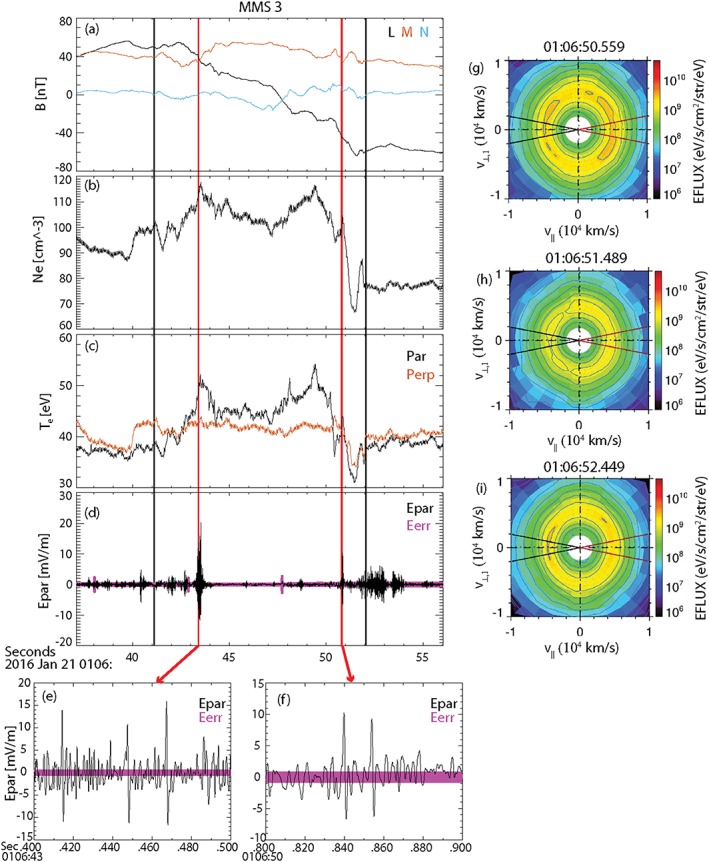
MMS 3 observations of (a) magnetic field, (b) electron density, (c) electron temperature, (d) parallel electric field, and (e and f) parallel electric field at the two times marked by red lines in panels (a)–(d). Note that the error in E_||_ is of the order 1 mV/m; (e)–(g) cuts of the electron differential energy flux in the plane defined by the magnetic field and the electron velocity, at three times before, during, and after the cavity. MMS = Magnetospheric Multiscale.

The electron heating and cooling can be explored by considering the fact that the electrons have a high thermal velocity and move rapidly along the magnetic field. This is illustrated by Figure 1c. The electron distributions above and below the reconnection exhaust are shown in red and blue, respectively. When the field line above the exhaust reconnects, the red antiparallel population is lost down the exhaust and is replaced by the blue population moving along the reconnected field line from below the current sheet. This passing population moves antiparallel to **B** and parallel to E_||_ and is decelerated. Furthermore, the lowest‐energy fraction of the blue population moving antiparallel to the field will be unable to cross the midplane. The consequence of this is both a decrease in *n*_*e*_ and *T*_*e*,par_. In contrast, when the field line below the current sheet reconnects, the blue parallel population is lost down the exhaust and is replaced by the red population from above the current sheet. This passing population is accelerated by E_||._ Since there is not a confining cavity, this contributes to the effective increase in *T*_*e*,par_ in the exhaust. MMS3 measurements of the electron differential energy flux before, during, and after the cavity encounter (Figures 4g–4i) show that in the cavity there is a depletion in the electron population moving antiparallel to **B**, in a manner consistent with this scenario and summarized in Figure 1c.

This implies that the change in electron temperature across the exhaust contains the signature of E_||_. In particular, the changes in the parallel temperature are of the order of 10 eV, from which a potential drop along the field line is ~10 V. If this were to be confined to a region of size comparable to 1 *d*_*i*_ (e.g., in the vicinity of the X‐line), then <E_||_ > ~0.4 mV/m. However, simulations indicate that E_||_ may also be strongly temporally and spatially structured in the cavity, with waves and instabilities (Drake et al., 2005). Figure 4d shows measurements of E_||_ at burst mode resolution. The strongest E_||_ signature is observed at 01:06:43.5 UT, the midpoint of the reversal in B_M_ when there is also a local maximum in *T*_*e*,par_ (Figure 4c). Figure 4e shows E_||_ in more detail at this time, revealing multiple isolated positive/negative bipolar signatures. These correspond to electron holes with diverging electric field structure moving in the +L direction along the magnetic field away from the X‐line. Hole signatures were not observed at all four spacecraft, placing limits on their size at the electron scale (electron inertial length *d*_*e*_ = 0.583 km). Figure 4d shows the cavity itself is notable for exhibiting very weak electric field signatures. Some confined E_||_ fluctuations are seen prior to the cavity encounter: Figure 4f shows that these also correspond to electron holes. We note that in simulations, holes have been similarly observed in the wall of the cavity, on the side adjacent to the exhaust (Markidis et al., 2012). This represents the first such observations in spacecraft data.

## 5 Summary

MMS observations show that during guide field reconnection, a pronounced density cavity confined to one edge of the exhaust exists ~100 *d*_*i*_ downstream from the X‐line, and also strong asymmetries in the Hall fields across the exhaust form. In the cavity, electron flow returning towards the X‐line is resolved for the first time. Furthermore, within the cavity electron cooling and enhanced ion parallel heating is resolved, thanks to the unprecedented resolution of the MMS measurements. This can be related to E_||_ and the fact that the electron thermal velocity is very high. When a field line reconnects, E_||_ slows down passing electrons moving into the cavity from the opposite side of the current sheet, reducing *n*_*e*_ and *T*_*e*_ and also resulting in a net electron flow towards the X‐line within the cavity. On the other hand, E_||_ appears to cause the acceleration of an ion beam in the cavity away from the X‐line. FPI electron and ion distributions support this interpretation.

The change in *T*_*e*_ gives an estimate of the potential drop and therefore E_||_. If averaged over the distance to the X‐line, E_||_ is below the limit of measurement. Alternatively, it could be the integrated effect of fluctuations, waves, and turbulence, but within the cavity, large fluctuations in E_||_ were not observed (although holes were present on the cavity edge). E_||_ could also be simply confined to the electron diffusion region at the X‐line, and observations made close to the X‐line (Wilder et al., 2017) suggest that E_||_ is sufficiently large to cause the observed temperature changes seen here 100 *d*_*i*_ downstream.

## References

[grl57340-bib-0001] Burch, J. L. , Moore, T. E. , Torbert, R. B. , & Giles, B. L. (2016). Magnetospheric Multiscale overview and science objectives. Space Science Reviews, 199(1‐4), 5–21. 10.1007/s11214-015-0164-9

[grl57340-bib-0002] Cattell, C. A. , Dombeck, J. J. , Wygant, J. , Drake, J. F. , Swisdak, M. M. , Goldstein, M. L. , et al. (2005). Cluster observations of electron holes in association with magnetotail reconnection and comparison to simulations. Journal of Geophysical Research, 110, A01211 10.1029/2004JA010519

[grl57340-bib-0003] Drake, J. F. , Shay, M. A. , Thongthai, W. , & Swisdak, M. (2005). Production of energetic electrons during magnetic reconnection. Physical Review Letters, 94(9), 095001 10.1103/PhysRevLett.94.095001 15783970

[grl57340-bib-0004] Drake, J. F. , & Swisdak, M. (2014). The onset of ion heating during magnetic reconnection with a strong guide field. Physics of Plasmas, 21(7), 072903 10.1063/1.4889871

[grl57340-bib-0005] Drake, J. F. , Swisdak, M. , Cattell, C. , Shay, M. A. , Rogers, B. N. , & Zeiler, A. (2003). Formation of electron holes and particle energization during magnetic reconnection. Science, 299(5608), 873–877. 10.1126/science.1080333 12574625

[grl57340-bib-0006] Drake, J. F. , Swisdak, M. , Phan, T. D. , Cassak, P. A. , Shay, M. A. , Lepri, S. T. , et al. (2009). Ion heating resulting from pickup in magnetic reconnection exhausts. Journal of Geophysical Research, 114, A05111 10.1029/2008JA013701

[grl57340-bib-0007] Eastwood, J. P. , Phan, T. D. , Oieroset, M. , Shay, M. A. , Malakit, K. , Swisdak, M. , et al. (2013). Influence of asymmetries and guide fields on the magnetic reconnection diffusion region in collisionless space plasmas. Plasma Physics and Controlled Fusion, 55(12), 124001 10.1088/0741-3335/55/12/124001

[grl57340-bib-0008] Eastwood, J. P. , Shay, M. A. , Phan, T. D. , & Øieroset, M. (2010). Asymmetry of the ion diffusion region Hall electric and magnetic fields during guide field reconnection: Observations and comparison with simulations. Physical Review Letters, 104(20), 205001 10.1103/PhysRevLett.104.205001 20867032

[grl57340-bib-0009] Ergun, R. E. , Tucker, S. , Westfall, J. , Goodrich, K. A. , Malaspina, D. M. , Summers, D. , et al. (2016). The axial double probe and fields signal processing for the MMS mission. Space Science Reviews, 199(1‐4), 167–188. 10.1007/s11214-014-0115-x

[grl57340-bib-0010] Fuselier, S. A. , & Lewis, W. S. (2011). Properties of near‐Earth magnetic reconnection from in‐situ observations. Space Science Reviews, 160(1‐4), 95–121. 10.1007/s11214-011-9820-x

[grl57340-bib-0011] Gosling, J. T. (2012). Magnetic reconnection in the solar wind. Space Science Reviews, 172(1‐4), 187–200. 10.1007/s11214-011-9747-2

[grl57340-bib-0012] Gosling, J. T. , & Phan, T. D. (2013). Magnetic reconnection in the solar wind at current sheets associated with extremely small field shear angles. The Astrophysical Journal Letters, 763(2), L39 10.1088/2041-8205/763/2/L39

[grl57340-bib-0013] Gosling, J. T. , Skoug, R. , McComas, D. J. , & Smith, C. W. (2005). Direct evidence for magnetic reconnection in the solar wind near 1 AU. Journal of Geophysical Research, 110, A01107 10.1029/2004JA010809

[grl57340-bib-0014] Horiuchi, R. , & Sato, T. (1997). Particle simulation study of collisionless driven reconnection in a sheared magnetic field. Physics of Plasmas, 4(2), 277–289. 10.1063/1.872088

[grl57340-bib-0015] Huba, J. D. (2005). Hall magnetic reconnection: Guide field dependence. Physics of Plasmas, 12(1), 012322 10.1063/1.1834592

[grl57340-bib-0016] Kleva, R. G. , Drake, J. F. , & Waelbroeck, F. L. (1995). Fast reconnection in high temperature plasmas. Physics of Plasmas, 2(1), 23–34. 10.1063/1.871095

[grl57340-bib-0017] Lindqvist, P.‐A. , Olsson, G. , Torbert, R. B. , King, B. , Granoff, M. , Rau, D. , et al. (2016). The spin‐plane double probe electric field instrument for MMS. Space Science Reviews, 199(1‐4), 137–165. 10.1007/s11214-014-0116-9

[grl57340-bib-0018] Markidis, S. , Lapenta, G. , Divin, A. , Goldman, M. , Newman, D. , & Andersson, L. (2012). Three dimensional density cavities in guide field collisionless magnetic reconnection. Physics of Plasmas, 19(3), 032119 10.1063/1.3697976

[grl57340-bib-0019] Mistry, R. , Eastwood, J. P. , Haggerty, C. C. , Shay, M. A. , Phan, T. D. , Hietala, H. , & Cassak, P. A. (2016). Observations of Hall reconnection physics far downstream of the X line. Physical Review Letters, 117(18), 185102 10.1103/PhysRevLett.117.185102 27835012

[grl57340-bib-0020] Mistry, R. , Eastwood, J. P. , Phan, T. D. , & Hietala, H. (2015). Development of bifurcated current sheets in solar wind reconnection exhausts. Geophysical Research Letters, 42, 10,513–10,520. 10.1002/2015GL066820

[grl57340-bib-0021] Mistry, R. , Eastwood, J. P. , Phan, T. D. , & Hietala, H. (2017). Statistical properties of solar wind reconnection exhausts. Journal of Geophysical Research: Space Physics, 122, 5895–5909. 10.1002/2017JA024032

[grl57340-bib-0022] Øieroset, M. , Phan, T. D. , Haggerty, C. , Shay, M. A. , Eastwood, J. P. , Gershman, D. J. , et al. (2016). MMS observations of large guide field symmetric reconnection between colliding reconnection jets at the center of a magnetic flux rope at the magnetopause. Geophysical Research Letters, 43, 5536–5544. 10.1002/2016GL069166

[grl57340-bib-0023] Øieroset, M. , Phan, T. D. , Shay, M. A. , Haggerty, C. C. , Fujimoto, M. , Angelopoulos, V. , et al. (2017). THEMIS multispacecraft observations of a reconnecting magnetosheath current sheet with symmetric boundary conditions and a large guide field. Geophysical Research Letters, 44, 7598–7606. 10.1002/2017GL074196

[grl57340-bib-0024] Paschmann, G. , Øieroset, M. , & Phan, T. (2013). In‐situ observations of reconnection in space. Space Science Reviews. 10.1007/s11214-012-9957-2

[grl57340-bib-0025] Phan, T. D. , Drake, J. F. , Shay, M. A. , Gosling, J. T. , Paschmann, G. , Eastwood, J. P. , et al. (2014). Ion bulk heating in magnetic reconnection exhausts at Earth's magnetopause: Dependence on the inflow Alfvén speed and magnetic shear angle. Geophysical Research Letters, 41, 7002–7010. 10.1002/2014GL061547

[grl57340-bib-0026] Phan, T. D. , Drake, J. F. , Shay, M. A. , Mozer, F. S. , & Eastwood, J. P. (2007). Evidence for an elongated (>60 ion skin depths) electron diffusion region during fast magnetic reconnection. Physical Review Letters, 99(25), 255002 10.1103/PhysRevLett.99.255002 18233527

[grl57340-bib-0027] Phan, T. D. , Eastwood, J. P. , Cassak, P. A. , Øieroset, M. , Gosling, J. T. , Gershman, D. J. , et al. (2016). MMS observations of electron‐scale filamentary currents in the reconnection exhaust and near the X line. Geophysical Research Letters, 43, 6060–6069. 10.1002/2016GL069212

[grl57340-bib-0028] Phan, T. D. , Gosling, J. T. , Paschmann, G. , Pasma, C. , Drake, J. F. , Oieroset, M. , et al. (2010). The dependence of magnetic reconnection on plasma beta and magnetic shear: Evidence from solar wind observations. Astrophysical Journal Letters, 719(2), L199–L203. 10.1088/2041-8205/719/2/l199

[grl57340-bib-0029] Phan, T. D. , Shay, M. A. , Gosling, J. T. , Fujimoto, M. , Drake, J. F. , Paschmann, G. , et al. (2013). Electron bulk heating in magnetic reconnection at Earth's magnetopause: Dependence on the inflow Alfven speed and magnetic shear. Geophysical Research Letters, 40, 4475–4480. 10.1002/grl.50917

[grl57340-bib-0030] Pollock, C. , Moore, T. , Jacques, A. , Burch, J. , Gliese, U. , Saito, Y. , et al. (2016). Fast plasma investigation for Magnetospheric Multiscale. Space Science Reviews, 199(1‐4), 331–406. 10.1007/s11214-016-0245-4

[grl57340-bib-0031] Pritchett, P. L. , & Coroniti, F. V. (2004). Three‐dimensional collisionless magnetic reconnection in the presence of a guide field. Journal of Geophysical Research, 109, A01220 10.1029/2003JA009999

[grl57340-bib-0032] Retinò, A. , Sundkvist, D. , Vaivads, A. , Mozer, F. , Andre, M. , & Owen, C. J. (2007). In situ evidence of magnetic reconnection in turbulent plasma. Nature Physics, 3(4), 235–238. 10.1038/nphys574

[grl57340-bib-0033] Robert, P. , Dunlop, M. W. , Roux, A. , & Chanteur, G. (1998). Accuracy of current density determination In Paschmann G. & Daly P. W. (Eds.), Analysis methods for multi‐spacecraft data (pp. 395–418). Bern: International Space Science Institute.

[grl57340-bib-0034] Russell, C. T. , Anderson, B. J. , Baumjohann, W. , Bromund, K. R. , Dearborn, D. , Fischer, D. , et al. (2016). The Magnetospheric Multiscale magnetometers. Space Science Reviews, 199(1‐4), 189–256. 10.1007/s11214-014-0057-3

[grl57340-bib-0035] Sonnerup, B. U. Ö. , & Scheible, M. (1998). Minimum and maximum variance analysis In Paschmann G. & Daly P. W. (Eds.), Analysis methods for multi‐spacecraft data (pp. 185–220). Bern: International Space Science Institute.

[grl57340-bib-0036] Torbert, R. B. , Russell, C. T. , Magnes, W. , Ergun, R. E. , Lindqvist, P. A. , LeContel, O. , et al. (2016). The FIELDS instrument suite on MMS: Scientific objectives, measurements, and data products. Space Science Reviews, 199(1‐4), 105–135. 10.1007/s11214-014-0109-8

[grl57340-bib-0037] Vörös, Z. , Yordanova, E. , Varsani, A. , Genestreti, K. J. , Khotyaintsev, Y. V. , Li, W. , et al. (2017). MMS observation of magnetic reconnection in the turbulent magnetosheath. Journal of Geophysical Research: Space Physics, 122, 11,442–11,467. 10.1002/2017JA024535

[grl57340-bib-0038] Wilder, F. D. , Ergun, R. E. , Eriksson, S. , Phan, T. D. , Burch, J. L. , Ahmadi, N. , et al. (2017). Multipoint measurements of the electron jet of symmetric magnetic reconnection with a moderate guide field. Physical Review Letters, 118(26), 265101 10.1103/PhysRevLett.118.265101 28707935

[grl57340-bib-0039] Yordanova, E. , Vörös, Z. , Varsani, A. , Graham, D. B. , Norgren, C. , Khotyaintsev, Y. V. , et al. (2016). Electron scale structures and magnetic reconnection signatures in the turbulent magnetosheath. Geophysical Research Letters, 43, 5969–5978. 10.1002/2016GL069191

